# Insights Into miRNA-mRNA Regulatory Mechanisms of Cold Adaptation in *Gymnocypris eckloni*: Ubiquitin-Mediated Proteolysis Is Pivotal for Adaptive Energy Metabolism

**DOI:** 10.3389/fgene.2022.903995

**Published:** 2022-07-22

**Authors:** Miaomiao Nie, Weilin Ni, Lihan Wang, Qiang Gao, Dan Liu, Fei Tian, Zhenji Wang, Cunfang Zhang, Delin Qi

**Affiliations:** ^1^ State Key Laboratory of Plateau Ecology and Agriculture, Qinghai University, Xining, China; ^2^ Key Laboratory of Adaptation and Evolution of Plateau Biota, Northwest Institute of Plateau Biology, Chinese Academy of Sciences, Xining, China; ^3^ Fishery Environmental Monitoring Station of Qinghai Province, Xining, China

**Keywords:** *Gymnocypris eckloni*, cold adaptation, MiRNA-mRNA, ubiquitin-mediated proteolysis, energy metabolism

## Abstract

This study aimed to understand cold stress adaptations mechanism in fish. Thus, the transcriptional response to cold conditions in *Gymnocypris eckloni* was evaluated using RNA-seq and microRNA (miRNA)-seq analyses. Low-temperature (LT) group *G. eckloni* was cultivated outdoors in waters cooled to 2–4°C for 3 weeks, while individuals in the control temperature (CT) group were exposed to 14–16°C. Significantly different responses were observed in both mRNA and miRNA expression profiles, with more mRNAs (1,833 and 1,869 mRNAs were up- and downregulated, respectively) and fewer miRNAs (15 and 6 were up- and downregulated, respectively) observed in the LT group individuals relative to the CT group individuals. A miRNA-mRNA network involved in the regulation of *G. eckloni* responses to cold stress was constructed; this network included ubiquitin-mediated proteolysis, protein processing, and oxidative phosphorylation. These results provided new insights into mechanisms of cold tolerance by fish, including decreased metabolic activity in addition to proteolysis.

## Introduction

Cold environments pose a significant challenge for many fish during winter because they inhibit growth (Yoneda and Wright 2005) and induce oxidative damage that inhibits cellular functioning (Heise et al., 2007; Wu et al., 2015), thereby resulting in other adverse effects. These challenges can decrease their feed intake and result in stagnation of growth and even catastrophic death, thus causing economic losses to companies or farmers. Thus, resolving cold-tolerance mechanisms can help improve cold-tolerance of fish and decrease winter-time aquaculture costs. Many studies have been conducted recently that primarily focus on polar (Chen et al., 2008; Coppe et al., 2013; Todgham et al., 2017; Blödorn et al., 2021) and model fish (Yang et al., 2011; Hu et al., 2016; Nitzan et al., 2019). In contrast, much less is known about other Schizothoracine fish species (Tong et al., 2017ab; Tong et al., 2017ab Zhou et al., 2020; Yang et al., 2021), thus confounding an understanding of their cold responses and cold adaptation metabolisms have developed a series of adaptation mechanisms.

One of the direct responses of organisms to cold conditions is transcriptional regulation. Cold-adapted fish like Antarctic fish depend on the regulation of several activities to adapt to cold conditions including protein processing, energy metabolism, immune system functioning, and signaling pathways. In particular, notothenioid fish exhibit diverse and unique adaptations to cold and highly oxygenated Antarctic marine waters. For example, compared to other fish, Antarctic notothenioid fish express more abundant Ub-conjugated proteins, which participated in maintaining proteins in cold and thermally stable native environments (Shin et al., 2012). Antarctic fish also altered the amino acid compositions to promoting protein fold and reducing protein denaturation because of cold temperatures (Berthelot et al., 2019). Moreover, loss of the heat shock response is also a strategy used for cold adaptation in fish (Bilyk et al., 2018). Moreover, Chen et al. (2008) identified that protein processing including biosynthesis, and degradation, antioxidation, lipid metabolism, and so on are functionally important for mitigating stresses because of freezing temperatures during notothenioid life histories. Researchers have paid considerable attention to cold adaptations in notothenioid fish, but additional studies are required to understand other adaptations to cold and hypoxic extreme environments.

Schizothoracine fish, the most diverse ichthyofauna taxa and restricted to the high-altitude environments, are well-adapted to Qinghai–Tibetan Plateau cold environments (Wu and Wu, 1992; Chen and Cao, 2000; Guan et al., 2014; Xia et al., 2016; Qi et al., 2018). Therefore, the fish represent excellent models that can be employed to understand the adaptive mechanisms of fish to LT (Qi et al., 2018). Genomic signature and comprehensive transcriptomic analyses in Schizothoracine fish have demonstrated that genes related to energy metabolism and transport have undergone positive selection and expansions that possibly underlie adaptations to the cold and hypoxic stresses (Tong et al., 2017ab). al., 2017ab *Gymnocypris eckloni* is a cold-water species, living in plateau waters of the upper reaches of the Yellow River (Wu and Wu, 1992; Chen and Cao, 2000). In these environments, fish play an important role in plateau freshwater ecosystems, as they are an important economic commodity in the Qinghai province. Most studies of *G. eckloni* have focused on their phylogenetics (Yang et al., 2017; Zhang et al., 2020) and hypoxia tolerance (Qi et al., 2018), while limited information is available regarding their adaptations to cold. In this study, miRNA, gene, and signaling pathway data were used to understand cold adaptations in *G. eckloni*, while the roles of miRNA-mRNA networks were revealed using high-throughput sequencing technology. These results provide a framework for understanding the regulatory mechanisms of fish cold tolerance.

## Materials and Methods

### Fish and Cold Acclimation

The experimental fish were collected from the Fishery Environmental Monitoring Station of Qinghai Province. Fish in the low-temperature (LT) group were cultivated in an outdoor pond with water temperatures cooled to 2–4°C in the winter. Thirty individuals randomly chosen from the outdoor pond were used in the control temperature (CT) group and acclimated at 14–16°C for 2 weeks in three tanks (0.2 m^3^). After 3 weeks, three individuals per group were immediately sampled following the anaesthetization with MS-222. Liver tissues were dissected, snap frozen, and stored in liquid nitrogen for further analysis.

### Preparation for RNA-Seq and Small RNA-Seq Libraries

Total RNAs from liver tissue were extracted using the Trizol Reagent (Invitrogen, CA, United States). A NanoDrop ND-1000 spectrophotometer (Nanodrop Technologies, Wilmington, DE, United States) was used to evaluate RNA concentration and purity. RNA integrity was calculated using the RNA Nano 6,000 Assay Kit of the Bioanalyzer 2,100 system (Agilent Technologies, CA, United States). High-quality RNAs libraries were subsequently used for RNA-seq and small RNA-seq.

A total amount of 3 µg RNA of each individual as experimental material were used for RNA sequencing. Sequencing libraries were obtained from the NEBNext^®^ Ultra^™^ RNA Library Prep Kit for Illumina^®^ (NEB, United States) according to the protocols and adding index adapters to delineate sequences from each library. In brief, poly-T oligo-attached magnetic beads were used for purified mRNA from total RNA. Fragmentation was then conducted using divalent cations with NEBNext First Strand Synthesis Reaction Buffer (5X) at high temperature. Random hexamer primers and M-MuLV Reverse Transcriptase (RNase H) were used for synthesizing first-strand cDNA. Thereafter, the synthesis of second-strand cDNA was achieved by RNase H and DNA Polymerase I. After the 3′ ends of DNA fragments were adenylated, NEBNext adapters were ligated with hairpin loop structures for hybridization. The AMPure XP beads system (Beckman Coulter) was using for the purification of library fragments, and cDNA fragments of 150–200 bp length were selected before sequencing on an Illumina NovaSeq 6,000 platform.

NEBNext^®^ Multiplex Small RNA Library Prep kit for Illumina^®^ (NEB, United States) was all set small RNA-seq libraries according to the instructions. Adding index adapters to delineate sequences from each sample. And Illumina HiSeq 2,500 platform using for small RNA sequencing.

### Quality Control and Annotation

We deposited raw RNA sequences into the NCBI Short Read Archive database (SRR19177535, SRR19177534, SRR19177533, SRR19177529, SRR19177528, SRR19177527, SRR19178221, SRR19178220, SRR19178219, SRR19178215, SRR19178214, and SRR19178213). These adapter sequences, those with >10% ambiguous “N” nucleotides, and low-quality sequences were removed using the software fastp (fastp -g -q 5 -u 50 -n 15 -l 150). The HISAT2 (2.0.5) software program was used to compare clean reads against the *G. eckloni* reference genome (unpublished data). Transcripts were identified using Trinity software splicing sequences, and the longest transcript was defined as unigene.

Homology annotation was conducted by subjecting non-redundant sequences to the NCBI (http://www.ncbi.nlm.nih.gov/) non-redundant protein (Nr), non-redundant nucleotide (Nt) databases, the Swiss-Prot database (http://www.ebi.ac.uk/uniprot/), GeneOntology (GO) database (http://www.geneontology.org/), Clusters of Orthologous Groups database (Arnaud et al., 2010) (http://www.ncbi.nlm.nih.gov/COG/), and Kyoto Encyclopedia of Genes and Genomes (KEGG) database (http://www.genome.jp/kegg/). If annotations from different databases conflicted, alignment analysis was used by comparing results from the above databases.

Clean data (clean reads) were acquired from removing the reads containing ploy-N and low-quality reads, and the adapter sequences were from raw data. The identify of known miRNAs by comparing small RNA tags against the miRbase20.0 reference databases. The modified software program mirdeep2 and srnatools-cli were used to obtain potential miRNAs and draw secondary structures.

Characteristic hairpin structures of miRNA precursors were forecasted using novel miRNAs by the miREvo and mirdeep2 software packages. The two software packages were integrated to explore dicer cleavage sites, secondary structures, and the minimum free energy of small RNA tags to forecasting novel miRNAs. Target genes of miRNAs were revealed by miRanda.

### Identification of Differentially Expressed Genes (DEGs), miRNAs (DEMs), and miRNA Targets

DEGs and DEMs between CT and LT groups were assessed based on the DESeq^R^ package (version 1.10.1 and version 1.8.3 for mRNA and miRNA targets, respectively). Furthermore, DEGs and miRNA targets were identified as those with an adjusted *p* < 0.05 and |log_2_(fold change)| > 0, while the threshold for DEMs was *p*_*adj*_ < 0.05. The functions of DEGs were displayed by GO categories, and KEGG was used for listed the biochemical pathways associated with RNAs.

### Quantification of DEGs and DEMs

The expression levels of mRNA and miRNA targets among liver samples were detected by real-time quantitative RT-PCR (qPCR). *β*-*actin* was selected as an internal control. These genes were randomly selected according to the sequencing data, and primers sequences were shown in Supplementary Table S1. The QPCR was performed on a LightCycler 480 PCR system using the SYBR Green PCR Master Mix (Takara, Dalian, Japan) with a program including denaturation at 95°C for 15 s, then 35 cycles of 5 s at 95°C, annealing at 60°C for 15 s, and extension for 25 s at 72°C.

### Statistical Analysis

Data are presented as means ± standard error. DEGs were identified between LT and CT groups using two-tailed *t*-tests. The threshold for significance was *p* < 0.05.

## Results

### Transcriptome Sequencing and Assembly

Separate RNA- and small RNA-seq libraries were constructed for each fish from the CT and LT groups. An approximate of 45 million (M) and 47 M raw RNA reads were generated for the CT and LT groups, respectively, corresponding to approximately 43 and 44 M clean reads (Supplementary Table S2). Among the above clean reads >74% were mapped to the reference genome, and we obtained a total of 23,903 unigenes from data (Supplementary Table S2). Small RNAs 18–35 nt in size were sequenced, which lead to the generation of approximately 11.1 and 11.8 M clean reads in the CT and LT groups, respectively (Supplementary Table S3). An analysis of reads length distribution showed that 21–23 nt was the most abundant miRNAs exhibited and comprised the majority of the miRNAs. In addition, the proportions of 21, 22, and 23 nt reads were higher in CT libraries (Supplementary Figure S1). An approximate of 289 known miRNAs and 240 novel mature miRNAs were identified, with a widespread distribution across miRNA families.

### DEGs and DEMs

**FIGURE 1:**
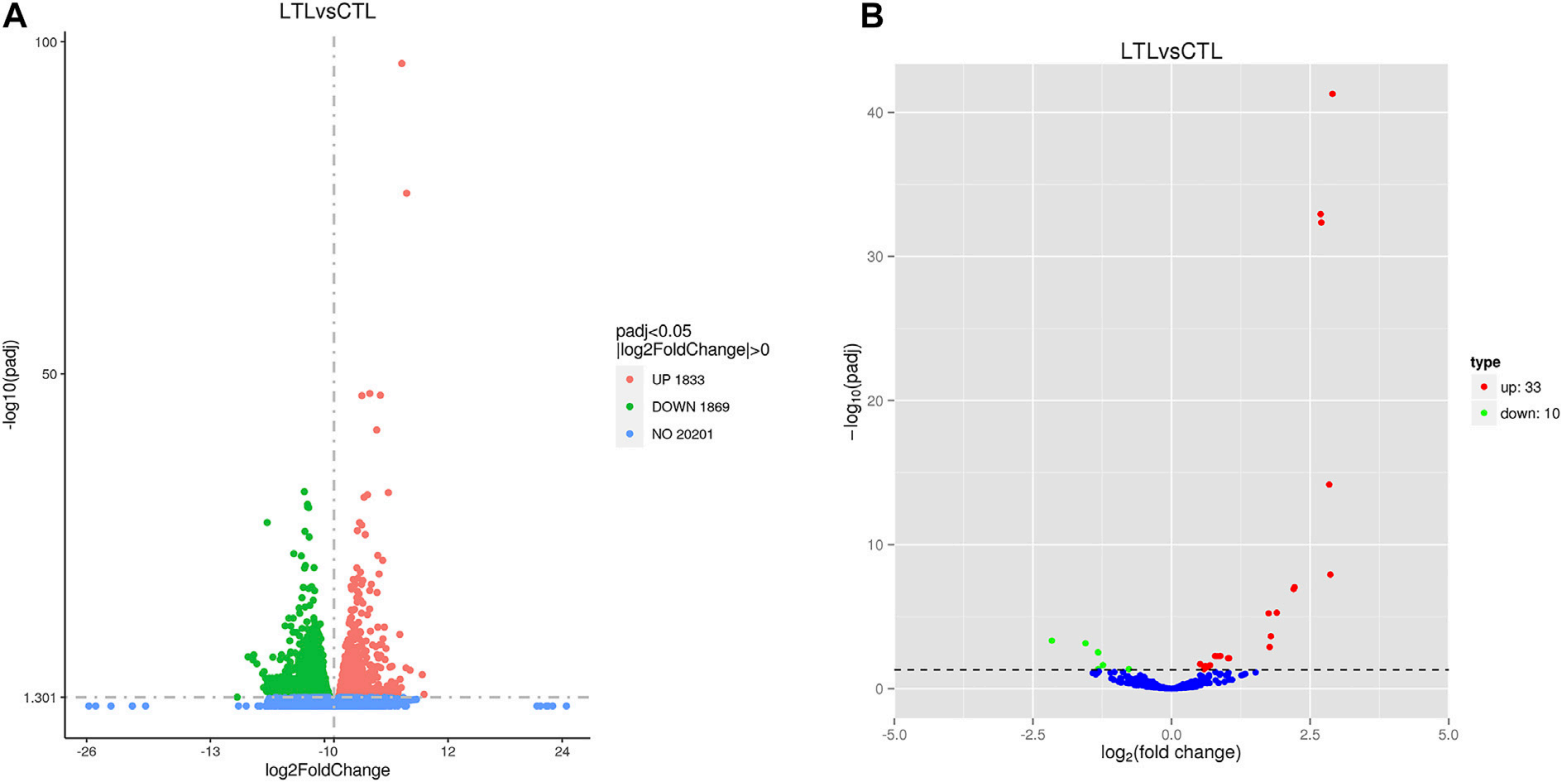
Volcano plots showing differentially expressed genes **(A)** and miRNAs **(B)** identified between the two treatment groups. Red and green circles indicate significantly upregulated and downregulated differences, respectively (*p* < 0.05), while blue circles indicate no differences.

The numbers of upregulated (1,833) and downregulated (1,869) genes in the LT group after cold acclimation were similar (Figure 1A). Relative to the CT group miRNA profiles, 33 upregulated miRNAs and 10 downregulated miRNAs were identified in the LT group (Figure 1B). Further inspection revealed that only 15 upregulated miRNAs and six downregulated miRNAs were present in the LT group because some RNAs represented identical repeats (Supplementary Table S4).

### Pathways Involving DEGs and miRNA-Targets

**FIGURE 2:**
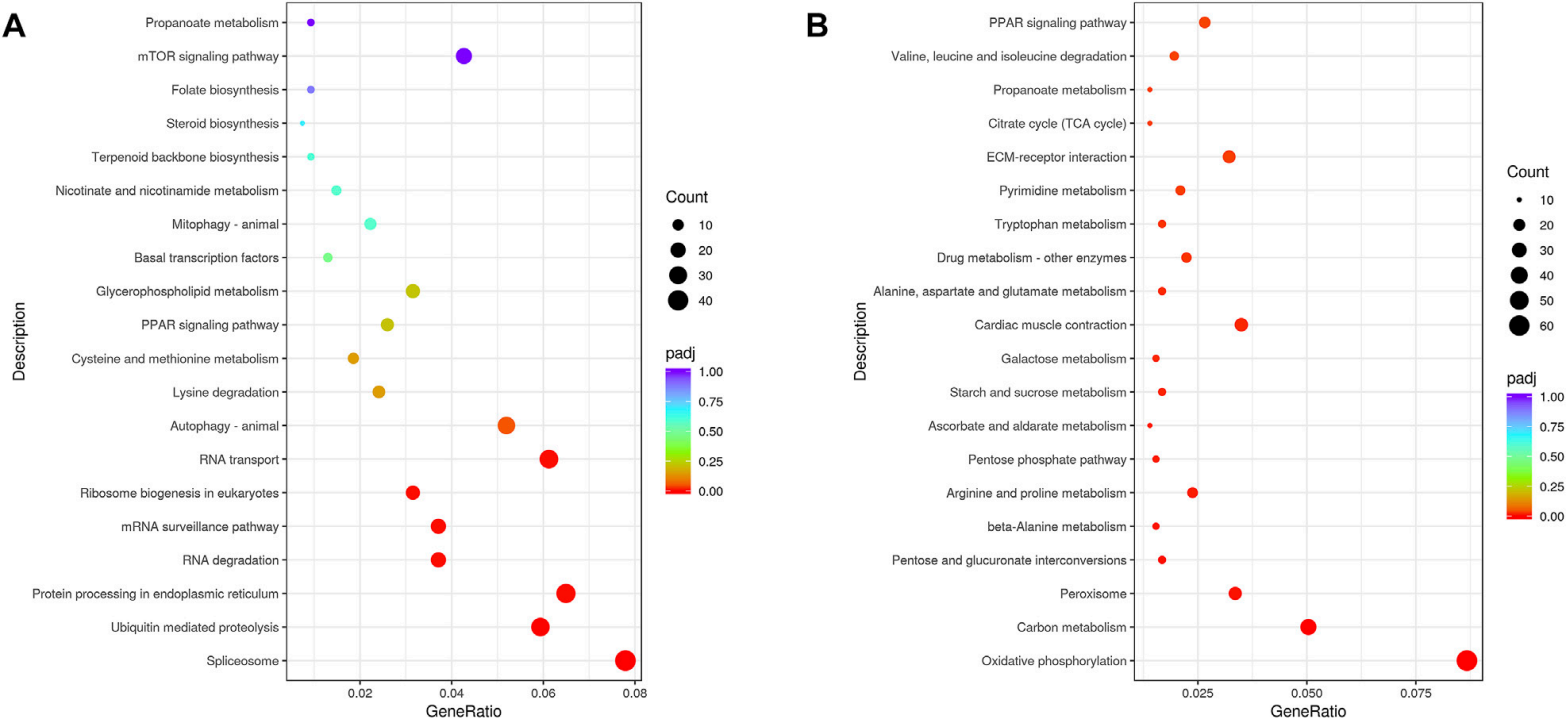
KEGG pathway classifications for upregulated **(A)** and downregulated **(B)** DEGs.

KEGG annotations were used to assign DEGs to specific physiological pathways (Figure 2). Upregulated DEGs for the LT vs. CT comparison were identified in the “ubiquitin-mediated proteolysis,” “spliceosome,” “protein processing in endoplasmic reticulum,” “RNA transport,” and “RNA degradation” pathways. In addition, downregulated DEGs were identified in the “oxidative phosphorylation,” “carbon metabolism,” “peroxisome,” “cardiac muscle contraction,” and “PPAR signaling pathway” pathways.

**FIGURE 3:**
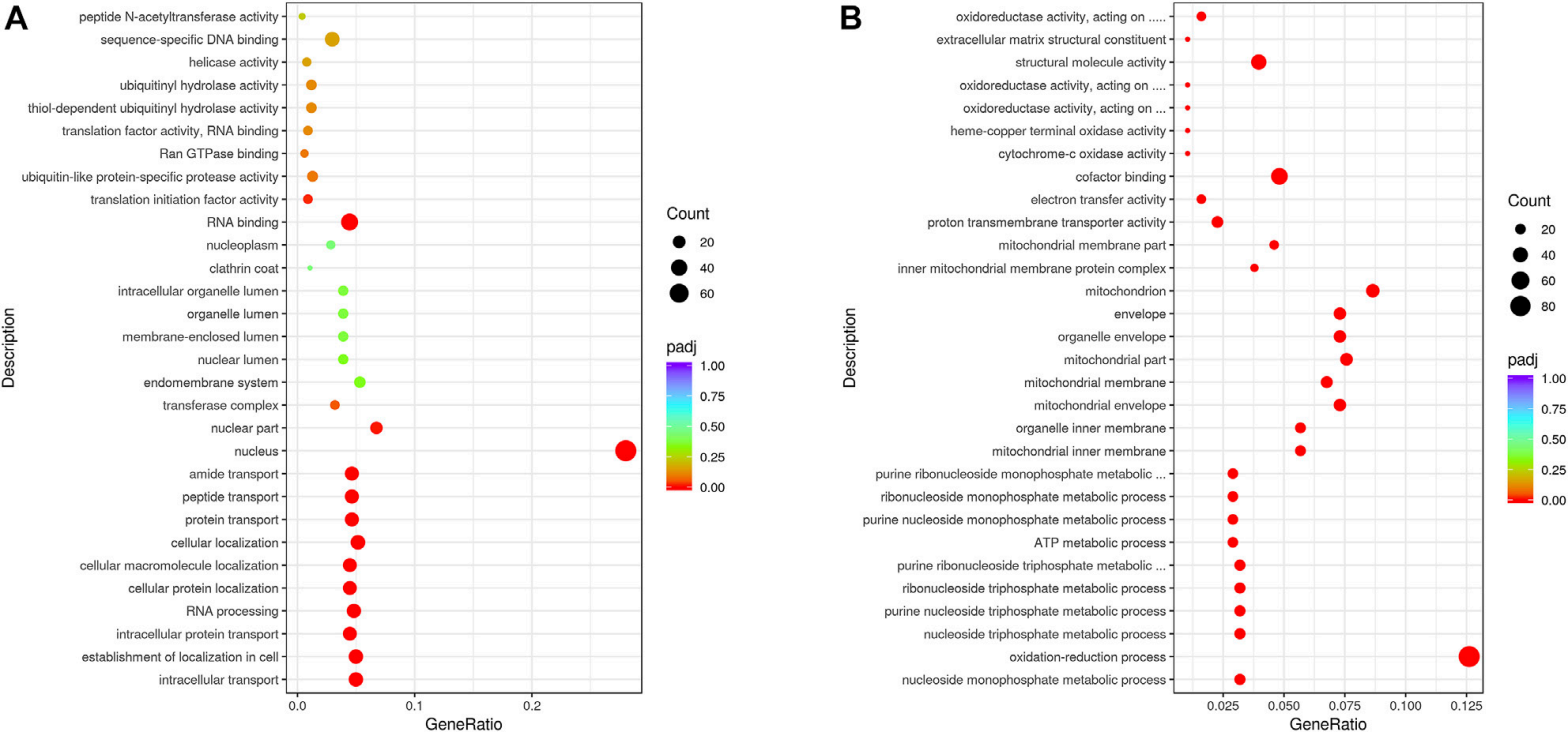
GO classifications of upregulated **(A)** and downregulated **(B)** DEGs.

The gene functions of upregulated DEGs were further analysis by GO, which revealed that they were primarily involved in biological processes, intracellular protein transport, RNA processing, nucleus functioning, and RNA binding. In addition, the functions of downregulated DEGs were primarily associated with the categories of biological processes (nucleoside monophosphate metabolic, oxidation-reduction, purine nucleoside triphosphate metabolic, and ribonucleoside triphosphate metabolic processes), cellular components (mitochondrial inner, organelle inner, and mitochondrial membranes), and molecular functions (cytochrome-c oxidase, electron transfer, cofactor binding, and oxidoreductase activities) (Figure 3). It is worth noting that low winter temperatures lead to *G. eckloni* decreasing their energy metabolism and reducing their physiological activities. We speculate that proteins are probably the primary source of energy for *G. eckloni*.

**FIGURE 4:**
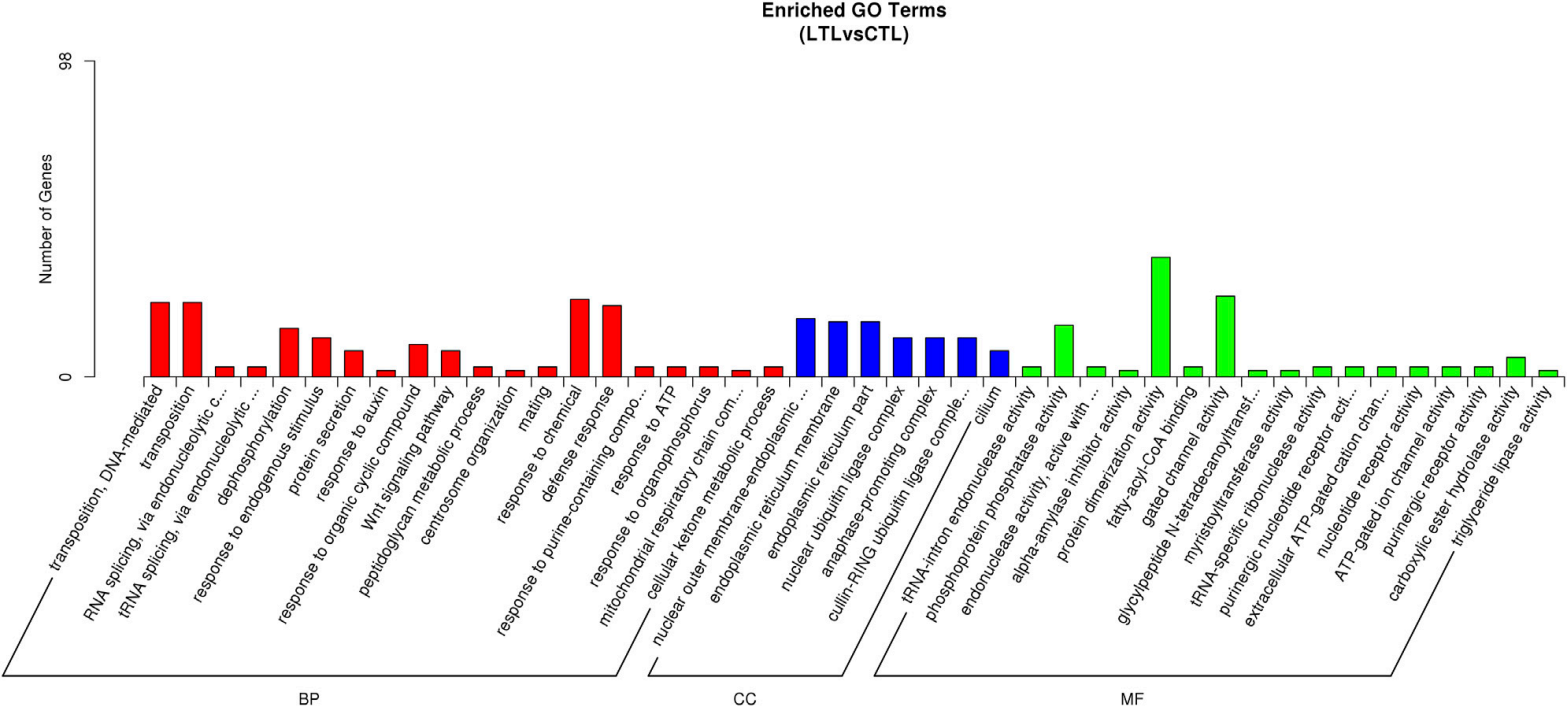
GO annotations of miRNA target genes.

An miRNA-targeted GO analysis of identified DEMs revealed that the miRNA targets were involved in biological processes including transposition (DNA-mediated), tRNA splicing (via endonucleolytic cleavage and ligation), dephosphorylation, responses to endogenous stimuli, protein secretions, the Wnt signaling pathway, peptidoglycan metabolic processes, cellular components including endoplasmic reticulum membranes, ubiquitin ligase complexes, and cilia, in addition to molecular functions including phosphoprotein phosphatase activity, endonuclease activity, purinergic nucleotide receptor activity, protein dimerization activity, fatty-acyl-CoA binding, tRNA-specific ribonuclease activity, and so on (Figure 4). KEGG pathways targeted by up- or downregulated miRNAs-included neuroactive ligand-receptor interactions, cell adhesion molecules, ubiquitin-mediated proteolysis, the Wnt and calcium signaling pathways, lysosomes, RNA transport, and amino-acid metabolism (Supplementary Figure S2).

### Integrated Analysis of DEGs and DEMs

The integrated analysis between miRNAs and mRNAs were drawn further. A total of 154 DEGs were found for the DEMs in the LT group compared to the CT group, which was defined as “intersection genes” according to Huang et al. (2015). Among these, 83 and 71 of the intersection genes were upregulated and downregulated, respectively. A total of 75 intersection genes in the LT group (38 and 37 upregulated and downregulated, respectively) were detected for the upregulated DEMs (miR-210-3p, miR-210b-5p, ola-miR-101b-5p, rno-miR-3574, miR-222a-5p, miR-29b-3p, miR-33a-5p, ipu-miR-29c, dre-miR-181a-2-3p, and ola-miR-181a-3p). A total of 83 intersection genes (34 and 49 upregulated and downregulated, respectively) were detected for the downregulated DEMs (mse-miR-2779, miR-143-5p, hsa-miR-375-3p, and novel_80) (Supplementary Table S5). A KEGG analysis suggested that the intersection genes were involved in ubiquitin-mediated proteolysis, biosynthesis of unsaturated fatty acids, fatty acid elongation, tyrosine metabolism, and ECM-receptor interactions (Supplementary Figure S3).

### Potential Cold Adaptation Markers and miRNA-mRNA Regulatory Networks

**FIGURE 5:**
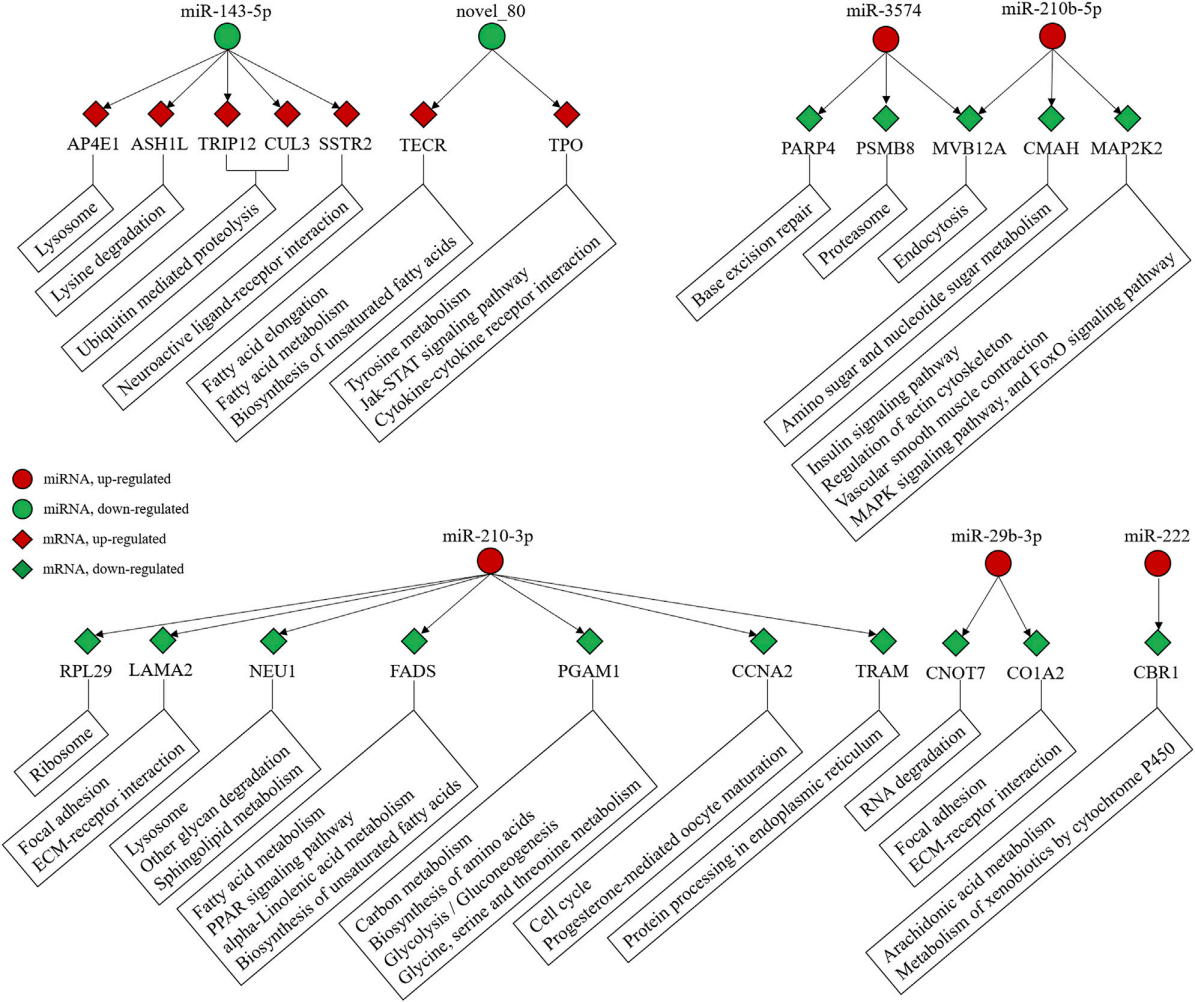
Interaction between miRNA-mRNA and their relative pathways.

Among the DEGs and DEMs, miR-143-5p, novel_80, miR-210-3p, miR-210b-5p, rno-miR-3574, miR-222, and miR-29b-3p may be involved in protein and fatty acid regulation during the winter. In particular, miR-143-5p regulates ubiquitin-mediated proteolysis via associated genes including *TRIP12* and *CUL3* (Figure 5 and Supplementary Table S6). Furthermore, the network was involved in protein, fatty acid, and sugar metabolism.

### Validation of RNA-Seq Data by qPCR

The expression of *ATP1B*, *HSP40*, *HSP70*, *SPAM1*, *GYS2*, *ST*S, *ApoB*, *SLC16A7*, and *eIF4E* (Supplementary Table S1) by qPCR to validate RNA-seq data. Our verification test indicated that the results of RT-qPCR were consistent with those of RNA-seq data (Supplementary Figure S4).

## Discussion

Responses to cold temperatures in cold environments are important as an adaptation for the life and growth of fish. Transcriptional regulation is a principal component of these responses (Chen et al., 2008; Mininni et al., 2014), while fewer studies have investigated the interactions between transcription and post-transcription under natural conditions. In the present study, fish at LT were cultivated outdoors in living environments very similar to their natural habitats to assess the transcriptional adaptations of *G. eckloni* to the LT during winter. These results indicate a principal transcriptional response that fish may be able to withstand cold conditions. Moreover, our results demonstrated that miRNA-mRNA networks were potentially important aspects of *G. eckloni* responses to cold native states.

Fish can remodel the process of life activity to alleviate cold damage to a different extent. This modification included signaling cascades and mitochondrial functioning, among others (Mininni et al., 2014). Similar changes were also observed in *G. eckloni* in this study. For example, *G. eckloni* significantly upregulated genes involved in the PPAR signaling pathway including *FATP*, *RXR*, *Apo-A1*, *ACS*, and *CPT-1*, genes involved in the mTOR signaling pathway such as *Wnt*, *eIF4E*, *S6K*, *PI3K*, and *GSK*, and genes involved in the FOXO signaling pathway such as *FOXO*, *SIRT1*, *G6PC*, and *IRS*. Some differences in the cold responses of *G. eckloni* were also observed relative to responses of other species. For example, the upregulated DEGs in LT vs. CT groups were involved in pathways including ‘‘spliceosome,” “ubiquitin-mediated proteolysis,” “RNA transport,” and “RNA degradation” pathways, with the functions of these genes being involved in intracellular protein transport, RNA processing, and RNA binding (Figures 2A, 3A). A previous analysis of gene evolution patterns within Schizothoracine fish suggested that the regulation of protein ubiquitination was an important adaptation to high altitude (Zhang et al., 2017). Furthermore, another study observed that temperature compensation of the ubiquitin–proteasome pathway in notothenioid fish led to better adaptation to cold (Todgham et al., 2017). In addition, downregulated DEGs identified in this study were involved in pathways including “oxidative phosphorylation,” “carbon metabolism,” “peroxisome,” “cardiac muscle contraction,” and the “PPAR signaling pathway,” with gene functions involved in mitochondrial membrane activity, oxidoreductase activity, and nucleoside metabolic processes (Figures 2B, 3B). Moreover, the posttranslational modification was more important for *G. eckloni* than in other species. In winter, *G. eckloni* decreases its energy metabolism to maintain the basic metabolic processes required to stay alive, while likely using protein as their main source of energy.

Studies that involved different species have been recently conducted to discover the roles of miRNAs in various life processes. Most studies of fish miRNA have focused on their function in the embryo development and adult life stages (Wienholds et al., 2003; Wienholds et al., 2005; Mishima 2012; Wei et al., 2012; Campos et al., 2014; Rasal et al., 2019; Bhat et al., 2021). MiRNA in cold-acclimated zebrafish (*Danio rerio*) might even act more in development rather than in thermal brain adaptation (Yang et al., 2011). Nevertheless, differentially expressed miRNAs (21) were detected during the adaptation to cold in this study, with more upregulated miRNAs (15) and less downregulated miRNAs (6) being observed. Among these miRNAs, the downregulated miRNAs miR-143-5p and miR-375 were particularly prominent. MiR-143 regulates adipocyte differentiation in the adipose tissue and also functions in the adipogenesis of rainbow trout *Oncorhynchus mykiss* (Esau et al., 2004; Mennigen et al., 2012). Further, miR-375 is pivotal for insulin-secreting island cells development in zebrafish (Kloosterman et al., 2007). In addition, miR-375 exerts an important role in proliferation, apoptosis, and cardiac morphogenesis of zebrafish embryos (Wang et al., 2016; Zhuang et al., 2020).

Under different temperature conditions, Senegalese sole *Solea senegalensis* documented the developmental plasticity of miRNA expression, with forecasted mRNA targets notably involved in the mTOR signaling pathway (Johnston et al., 2009). In this study, the upregulated miRNAs, miR-181-3p, miR-33, and miR-146 were prominent. During the segmentation, miR-181-3p was preferentially expressed at 15°C relative to that at 21°C, and it negatively regulates myogenesis by the target, calpain (Campos et al., 2014). Furthermore, miR-181a also exhibits multiple roles in cell proliferation and invasion, as well as in immune regulation (Li et al., 2007; Xu et al., 2016; Chiachanpongse et al., 2018; Woldemariam et al., 2020; Barth et al., 2021). What is even more important, miRNAs can remodel protein abundances and the phosphorylation status of key proteins in the insulin pathway. For example, the expression levels of miR-122 (Esau et al., 2006) and miR-33 (Rayner et al., 2010) were inversely correlated with the expression and phosphorylation level of the α-subunit of AMPK (adenosine 5‘-monophosphate (AMP)-activated protein kinase). It is also worth noting that miR-181a and miR-222 exhibit different expression patterns under high temperature treatments (unpublished data) compared to low temperature stress. As a result, miR-miR-181a and miR-222 could be potential markers of thermal adaptation worthy of future analysis.

Previous studies of teleost miRNAs have focused primarily on their development and energy metabolism (Bizuayehu and Babiak, 2014; Mennigen, 2016). However, studies have confirmed that miRNA-dependent regulation existed in both lipid and carbohydrate metabolism (Flowers et al., 2013; Hinault et al., 2013), in addition to coordinated interaction of both pathways (Lynn, 2009). Many metabolic pathways are highly regulated at the gene expression level; thereby, the protuberant role of miRNAs in metabolism is necessary (Desvergne et al., 2006). Nevertheless, miRNAs roles should be evaluated in future studies because they may act as important regulators in fish adaptations to cold.

## Conclusion

To the best of our knowledge, this was the first study that showed potential miRNA-mRNA networks involved in the response of Schizothoracine fish to cold adaptations. Following its adaptation to cold, *G. eckloni* exhibited better responses at the transcriptional and post-transcriptional levels via miRNA-mRNA interactions that targeted various pathways. These regulatory dynamics could impact various biological processes, for example, inducing genes expression involved in ubiquitin-mediated proteolysis, protein processing, and oxidative phosphorylation, among other pathways. Overall, these results suggested that decreased metabolic activities—in addition to widespread transcriptomic responses—could explain the ability of *G. eckloni* to survive at lower temperatures. Furthermore, these results suggested that miR-101b-5p, miR-29b-3p, miR-2779, miR-181a, and miR-222 could be potential thermal markers worthy of future research. However, the regulatory network of miRNA and their targets involved in cold adaptation process remain indistinct and require further studies.

## Data Availability

The datasets presented in this study can be found in online repositories. The names of the repository/repositories and accession number(s) can be found in the article/Supplementary Material.
