# Supplementary material for: Insights Into miRNA-mRNA Regulatory Mechanisms of Cold Adaptation in Gymnocypris eckloni: Ubiquitin-Mediated Proteolysis Is Pivotal for Adaptive Energy Metabolism
Source: Front Genet. 2022 Jul 22;13:903995. doi: 10.3389/fgene.2022.903995 (PMC9354851; doi:10.3389/fgene.2022.903995)
Supplement: Supplementary file 2 [file Table1.docx]

Insights into miRNA-mRNA regulatory mechanisms of cold adaptation in *Gymnocypris eckloni*: Ubiquitin-mediated proteolysis is pivotal for adaptive energy metabolism

Miaomiao Nie^1^, Weilin Ni^1^, Lihan Wang^1^, Qiang Gao, Dan Liu^1^, Fei Tian^2^, Zhenji Wang^3^, Cunfang Zhang^1^, Delin Qi^1∗^

^1^State Key Laboratory of Plateau Ecology and Agriculture, Qinghai University, Xining 810016, China. ^2^ Key Laboratory of Adaptation and Evolution of Plateau Biota, Northwest Institute of Plateau Biology, Chinese Academy of Sciences, Xining 810001, China. ^3^Fishery Environmental Monitoring Station of Qinghai Province, Xining, 810012, China

* **Correspondence:**

Corresponding Author: Delin Qi, delinqi@126.com

**Table S1** Genes and specific primers used for qPCR

**Table S2** Data output quality list of mRNAs

**Table S3** Data output quality list of small RNA

**Table S4** The DEMs of LTL vs. CTL

**Table S5** Integrated analysis of DEMs and DEGs

**Table S6** miRNA-mRNA and the relative pathway

Table S1 Genes and specific primers used for qPCR

| Gene Name | | Sequence (5’--3’) |
| --- | --- | --- |
| *SPAM1* | -F | AATTCCAGCAAGAAGCCAGA |
|  | -R | TGCTTCCACTCACAGAAACG |
| *GYS2* | -F | CCTGCCAAAACCAACAACTT |
|  | -R | TTGCATAAATGGCCCTCTTC |
| *STS* | -F | GGATTCGGATACCAGGGATT |
|  | -R | TGTTCAGAACGCTCCACTTG |
| *ApoB* | -F | CGGAGACTCTGGACTTGAGG |
|  | -R | TCCATGCCAATCTCAATGAA |
| *SLC16A7* | -F | CCCCAAATCCCTTACCATCT |
|  | -R | AATGGTGGTGCCAAAAGAAG |
| *HSP70* | -F | CAACGGCAGAGACTTGAACA |
|  | -R | AGGTCTGGGTCTGTTTGGTG |
| *eIF4E* | -F | AATAAGACGGCCAGTGACCA |
|  | -R | GCCTCCAGTTGCATTCACAA |
| *HSP40* | -F | CTGGGGATAAAGAAAGGCGC |
|  | -R | TGAAATTACCACCTCCGCCT |
| *ATP1B* | -F | ATGACGATGGAGTGCAGGAC |
|  | -R | GGCAGATACCCGAGAATCCG |
| *β-actin* | -F | GAACCCCAAGGCTAACAGAGAA |
|  | -R | AGGCATACAGGGACAGCACA |

Table S2 Data output quality list of mRNA

| Sample | Raw Reads | Clean reads | Clean bases | Error (%) | Q20 (%) | Q30 (%) | Total map |
| --- | --- | --- | --- | --- | --- | --- | --- |
| LTL1 | 45204270 | 43433084 | 6.51G | 0.02 | 98.78 | 96.01 | 34518537(74.75%) |
| LTL2 | 44660010 | 42804320 | 6.42G | 0.02 | 98.81 | 96.08 | 33368407(74.26%) |
| LTL3 | 45921590 | 44146328 | 6.62G | 0.02 | 98.66 | 95.58 | 31148339(74.73%) |
| CTL1 | 48286210 | 46177656 | 6.93G | 0.02 | 98.75 | 95.94 | 33934113(78.13%) |
| CTL2 | 48123310 | 44936728 | 6.74G | 0.02 | 98.77 | 95.95 | 32228330(75.29%) |
| CTL3 | 44434438 | 41682858 | 6.25G | 0.02 | 98.84 | 96.16 | 33879021(76.74%) |

Q20/Q30: the percentage of base, whose Phred values greater than 20 / 30; Q_phred_=-10log_10_(e).

Table S3 Data output quality list of small RNA

| Sample | Total reads | Clean reads | Bases (G) | Error rate (%) | Q20 (%) | Q30 (%) | GC (%) |
| --- | --- | --- | --- | --- | --- | --- | --- |
| LTL1 | 12530340 (100.00%) | 12213328 (97.47%) | 0.627 | 0.01 | 99.46 | 97.81 | 49.77 |
| LTL2 | 12574236 (100.00%) | 12255222 (97.46%) | 0.629 | 0.01 | 99.21 | 97.01 | 49.65 |
| LTL3 | 11694182 (100.00%) | 10806187 (92.41%) | 0.585 | 0.01 | 99.43 | 97.79 | 50.21 |
| CTL1 | 11322141 (100.00%) | 10319076 (91.14%) | 0.566 | 0.01 | 99.50 | 97.96 | 50.16 |
| CTL2 | 12850052 (100.00%) | 12490489 (97.20%) | 0.643 | 0.01 | 99.50 | 97.97 | 50.34 |
| CTL3 | 11578295 (100.00%) | 10352581 (89.41%) | 0.579 | 0.01 | 99.42 | 97.63 | 50.19 |
